# Accuracy of dynamic computer-assisted implant surgery among novice operators using three different patient registration methods: an in-vitro study

**DOI:** 10.1186/s40729-026-00666-6

**Published:** 2026-03-31

**Authors:** Ryo Yamamoto, Toshiki Nojiri, Daichi Yoshida, Yutaro Oyamada, Akihiro Fukutoku, Kazuhiro Kon

**Affiliations:** Division of Fixed Prosthodontics and Oral Implantology, Department of Prosthodontics, School of Dentistry, Iwate Medical University, Morioka, Japan

**Keywords:** Dynamic navigation, Computer-assisted implant surgery, Accuracy

## Abstract

**Introduction:**

Accurate implant placement with computer-assisted implant surgery (CAIS) is critical to ensuring long-term success. Dynamic computer-assisted implant surgery (d-CAIS) enhances surgical precision through real-time feedback; however, its accuracy and procedural efficiency when used by novice operators remain insufficiently investigated. This study evaluated how different registration methods in d-CAIS influence implant placement accuracy and procedural time when performed by novice operators.

**Materials and methods:**

In this in vitro study, three registration methods were assessed (surface-based registration: ND, marker-based registration: XC, and pair-point registration: XM groups). Five novice operators, defined as dentists with less than five years of implant experience, placed 25 implants per group (75 total) in partially edentulous maxillary models. Postoperative cone-beam computed tomography scans were used to measure deviations at the implant entry point, apex, vertical depth, and angle. Statistical analyses were performed to compare group differences.

**Results:**

Mean three-dimensional deviations at the implant entry point were 0.97 mm (ND), 0.68 mm (XC), and 0.72 mm (XM); at the apex: 1.33 mm, 0.78 mm, and 0.90 mm, respectively. Vertical depth deviations at the apex were comparable across groups: 0.62 mm (ND), 0.51 mm (XC), and 0.54 mm (XM). Angular deviation was highest in the ND group (3.16°) compared to the XC (1.18°) and XM (0.97°) groups, with significant differences observed between ND and both XC and XM. Average procedural time was longest in the ND group and shortest in the XM group, with statistically significant differences between ND and the other two groups.

**Conclusions:**

In novice operators, d-CAIS enabled stable control of implant entry point position and insertion depth regardless of the registration method used. In contrast, angular accuracy and procedural efficiency were influenced by registration strategies and system-related characteristics. These findings suggest that selection of appropriate registration methods may play an important role in optimizing accuracy and workflow efficiency during the early learning phase of d-CAIS.

## Introduction

Dental implant therapy has been widely reported to demonstrate high long-term survival rates and favorable functional outcomes, and is therefore considered a primary treatment option for the rehabilitation of partially or completely edentulous patients [1, 2]. Implant placement is frequently performed in close proximity to critical anatomical structures, such as the inferior alveolar nerve, the maxillary sinus, and major vascular pathways in the floor of the mouth, making highly accurate positioning essential to avoid inadvertent surgical complications [3–6]. Furthermore, with the widespread adoption of prosthetically driven treatment concepts, even minor deviations in implant position may influence not only long-term clinical outcomes but also esthetic results, thereby increasing the demand for greater precision in implant placement [7–9].

Against this background, computer-assisted implant surgery (Computer-Assisted Implant Surgery: CAIS) has rapidly evolved in recent years [10]. CAIS integrates three-dimensional digital information derived from cone-beam computed tomography (CBCT) and optical scanning to transfer preoperative implant planning to the surgical field with a high degree of accuracy, and its clinical applications have expanded substantially over the past decade [11]. Two principal approaches to CAIS exist: a static approach using surgical guides (static CAIS: s-CAIS) and a dynamic approach that provides real-time positional feedback during surgery (dynamic CAIS: d-CAIS) [10]. Although s-CAIS has been shown to provide consistent and reliable accuracy [12, 13], it is associated with several limitations, including restricted surgical visibility, reduced tactile feedback, limited flexibility for intraoperative plan modification, and challenges in cases with limited intraoral space [14, 15]. In contrast, d-CAIS allows continuous monitoring of the position, angulation, and depth of surgical instruments and jaw anatomy through optical tracking systems, without the need for bulky intraoral guiding devices, thereby facilitating effective irrigation and improved operability [16, 17]. Moreover, d-CAIS has been reported to offer greater intraoperative flexibility than static approaches, as surgical plans can be adjusted in response to unexpected anatomical findings encountered during the procedure [18]. Beyond its clinical advantages, d-CAIS has attracted increasing attention for its potential educational benefits, particularly as a training tool for less-experienced clinicians. Real-time visual feedback enables operators to recognize deviations in their hand movements and angulation control, which may contribute to a shortened learning curve [19–21]. However, the extent to which these educational benefits are realized in practice may vary considerably depending on the operator’s level of experience [22, 23].

Although multiple factors can influence the accuracy of d-CAIS, patient registration, which aligns preoperative planning data with the patient’s actual anatomy, represents one of the most critical steps in the digital workflow. Registration methods vary among navigation systems and commonly include surface-based registration, in which dental surface morphology is captured as point clouds (e.g., Navident®); pair-point registration, which relies on clearly defined anatomical landmarks for coordinate matching (e.g., X-Mark); and marker-based registration, in which radiopaque fiducial markers are used to align CBCT data with intraoperative coordinates (e.g., X-Clip) [10]. Because these registration techniques differ in terms of data acquisition complexity, the number of operational steps required, and reproducibility, it has been suggested that their impact on placement accuracy may vary depending on the operator’s level of skill and experience.

In particular, among less-experienced operators, insufficient understanding of the registration steps and a lack of procedural fluency are more likely to manifest as measurable errors. While experienced clinicians are generally able to select and apply multiple registration methods appropriately, novice operators may be more susceptible to increased cognitive load and procedural uncertainty, which can directly translate into final implant placement deviations. Despite this, there is a paucity of studies specifically investigating which registration method is most user-friendly and least prone to error for novice operators. Moreover, the majority of previous studies have focused primarily on accuracy comparisons performed by experienced clinicians [24, 25], and a certain body of evidence has accumulated regarding the extent to which differences in registration methods influence clinical outcomes. However, no studies to date have systematically evaluated the effects of different registration strategies on error patterns, procedural burden, and learning curves in clinicians with less than five years of implant experience. This gap in the literature may represent a significant barrier to the widespread implementation of d-CAIS in educational settings and routine clinical practice. From the perspective of structured training for novice operators, it is critically important to understand which registration methods facilitate more stable accuracy and in which aspects of implant placement errors are most likely to occur. Registration strategies should not be regarded merely as system-dependent technical differences; rather, they represent factors that directly influence the operator’s cognitive processing and spatial perception. Consequently, the most suitable registration method for novice operators may differ from that for experienced clinicians. Therefore, it may not be appropriate to directly extrapolate findings derived from studies involving experienced operators to novice clinicians. Instead, dedicated investigations focusing on error characteristics unique to novice operators are required to support the safe and effective adoption of d-CAIS during the early stages of clinical training [19, 21, 24].

Based on the aforementioned background, the present study aimed to evaluate the influence of three major patient registration strategies—(1) surface-based, (2) marker-based, and (3) pair-point–based registration—on implant placement accuracy and procedural efficiency in d-CAIS, specifically among clinicians with less than five years of implant experience, using a standardized in vitro experimental model. Unlike previous studies that primarily focused on experienced operators, the present investigation is unique in that it seeks to elucidate error profiles and method-specific compatibilities that are characteristic of clinicians in the learning phase. The findings of this study are expected to contribute to the evaluation of the educational potential of d-CAIS, inform system selection during early clinical adoption, and support the optimization of learning curves. Ultimately, these insights may facilitate improvements in procedural safety and implant placement accuracy in future clinical practice.

## Materials and methods

### Study design

This study was designed as an in vitro experimental investigation conducted at the Department of Prosthodontics and Oral Implantology, School of Dentistry, Iwate Medical University. The purpose of the study was to evaluate the effects of three different patient registration methods used in d-CAIS on implant placement accuracy and procedural efficiency. The registration methods evaluated were surface-based registration using the Navident® system (ND group), radiographic marker–based registration using the X-Clip method with the X-Guide® system (XC group), and pair-point registration using the X-Mark method with the X-Guide® system (XM group). The primary objective of this study was to compare the effects of these different registration methods on the accuracy and procedural time of d-CAIS when performed by novice operators with limited implant placement experience.

### Operators

In this study, novice operators were defined as dentists who met all of the following criteria: less than five years of experience in implant placement, a cumulative number of fewer than 30 implant placements, and no or only minimal clinical experience with d-CAIS. A total of five dentists who fulfilled these criteria participated in the study.

### Preoperative training and standardization of procedures

To minimize inter-operator variability among novice operators, preoperative training and procedural workflows were standardized. For all cases, software-related tasks—including the import of CBCT and STL data, digital planning of implant placement, and initial setup of the navigation system—were performed by an experienced operator with extensive expertise in d-CAIS. In contrast, all hands-on procedures from patient registration through implant placement were performed by the novice operators. This study design allowed the exclusion of variability related to planning and software manipulation while enabling the assessment of novice-specific effects associated with registration workflows and implant placement procedures. Prior to the experiment, standardized instructions were provided for all three registration workflows to ensure an equivalent level of procedural understanding among operators. These instructions were limited to the fundamental concepts, operational steps, and critical considerations of each registration method, and no guidance favoring any specific technique was provided. No trial or practice implant placements were allowed before the experiment, and all novice operators commenced the procedures under identical conditions.

### Control of order effect

To account for the influence of learning curves among novice operators, the potential order effect associated with the sequence of registration methods was controlled. The order in which the three registration methods (ND, XC, and XM) were performed was randomized for each operator according to a predefined allocation table. Each operator performed five consecutive implant placements using each registration method. This consecutive design was considered to exert a uniform learning effect across all operators and registration methods.

### Data acquisition

In this study, a total of 75 partially edentulous maxillary models were used. All models underwent CBCT scanning (KaVo OP 3D Vision, KaVo Dental GmbH, Germany), and DICOM data were obtained. For the XC group, the X-Clip was fitted to each model prior to CBCT acquisition, positioned to cover the region from the right maxillary central incisor to the canine, and CBCT imaging was performed with the marker in place. In addition, surface morphology of all models was captured using an intraoral scanner (Trios 4; 3Shape, Copenhagen, Denmark), and the data were exported as STL files.

### Data registration and implant placement

**Fig. 1:**
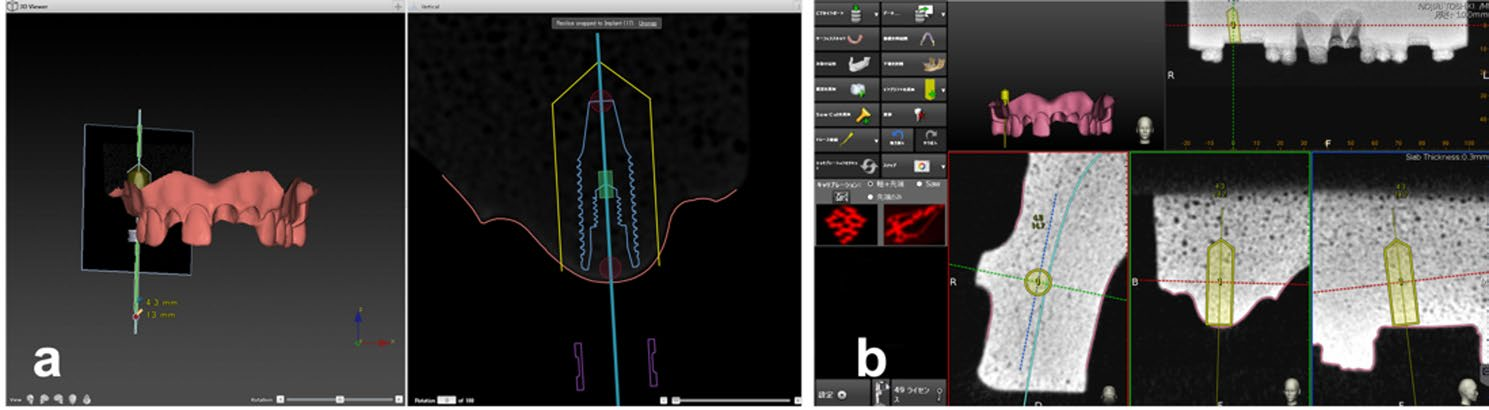
Implant placement planning for each system: **a** Nobel DTX Studio®; **b** Navident®

**Fig. 2:**
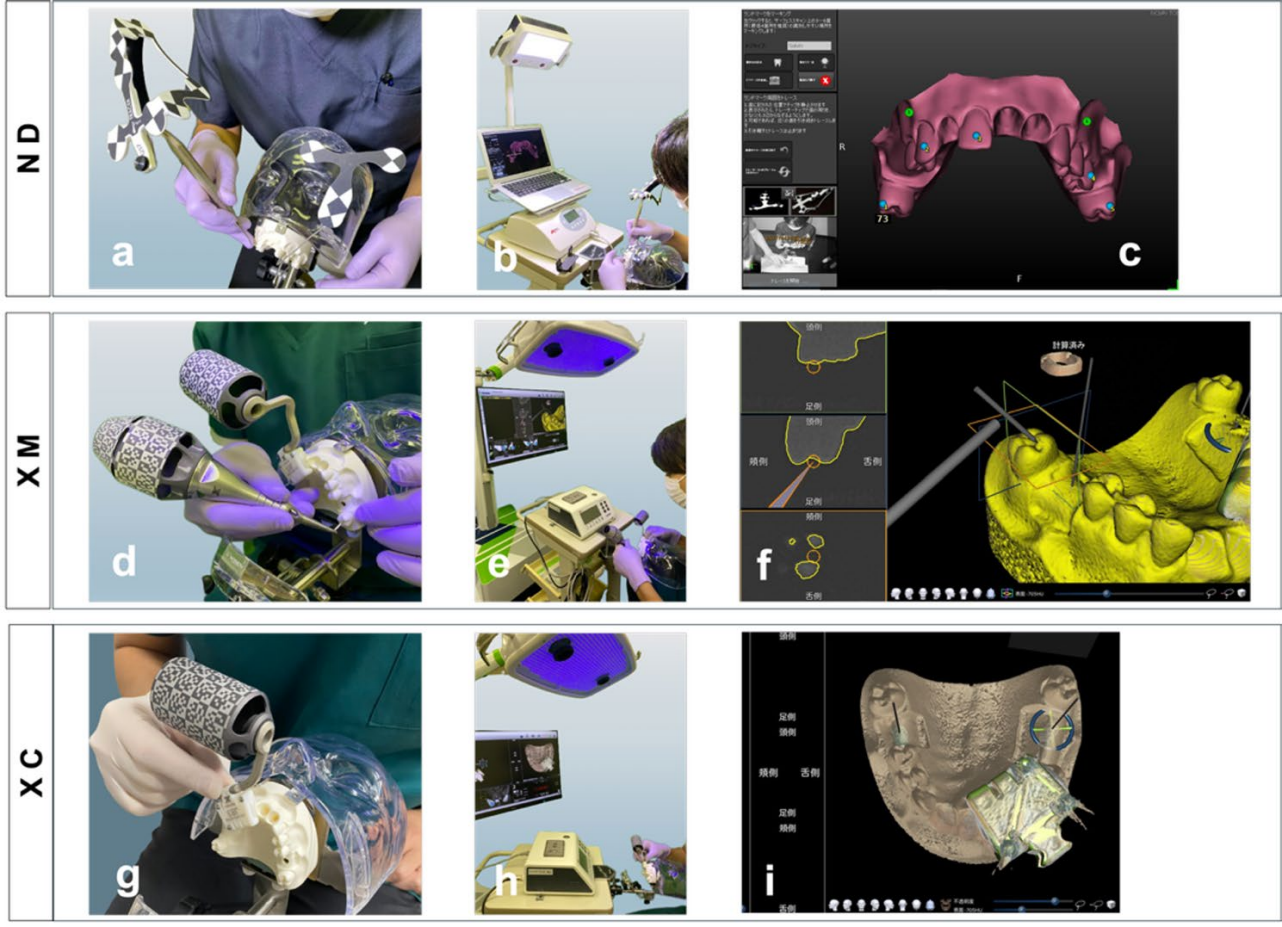
Registration workflows for each d-CAIS system. **a** In the ND group, the tracer traces the hard tissue surface. **b** The optical tracking system captures the movement of the tracer, and the software recognizes the surface morphology of the hard tissue. **c** Monitor view during the registration process. **d** In the XM group, the X-Mark probe tool traces the hard tissue surface. **e** The optical tracking system captures the position of the X-Mark probe tool, and the software recognizes the surface morphology of the hard tissue. **f** Monitor view during the registration process. **g** In the XC group, the X-Clip is attached to the dentition. **h**, **i** The optical tracking system recognizes the fiducial markers (radiopaque metal balls) attached to the X-Clip, and the software automatically performs registration

The acquired DICOM and STL data were imported into the dedicated software of each navigation system. For all cases, a single experienced operator with extensive expertise in d-CAIS created a standardized implant placement plan for the edentulous site at the right maxillary first molar region (Fig. 1). Each model was mounted in a phantom head, and all procedures were performed under conditions simulating the clinical environment. Registration and implant placement were performed exclusively by the novice operators. Registration procedures were conducted in accordance with the manufacturer-recommended protocols for each system. In the ND group, surface-based registration was performed by tracing the dental surface morphology. In the XM group, pair-point registration was carried out using multiple clearly defined anatomical landmarks. In the XC group, radiographic marker–based registration was performed using the X-Clip positioned in the same location as during CBCT acquisition (Fig. 2). After completion of registration, calibration of the drill axis and drill tip was performed. Osteotomy preparation and implant placement were then conducted under d-CAIS guidance at the edentulous site of the right maxillary first molar region. The implants used were Nobel Parallel CC RP implants (diameter 4.3 mm, length 13 mm). Procedural time was recorded as the duration from completion of registration to final implant placement.

### Accuracy assessment

**Fig. 3:**
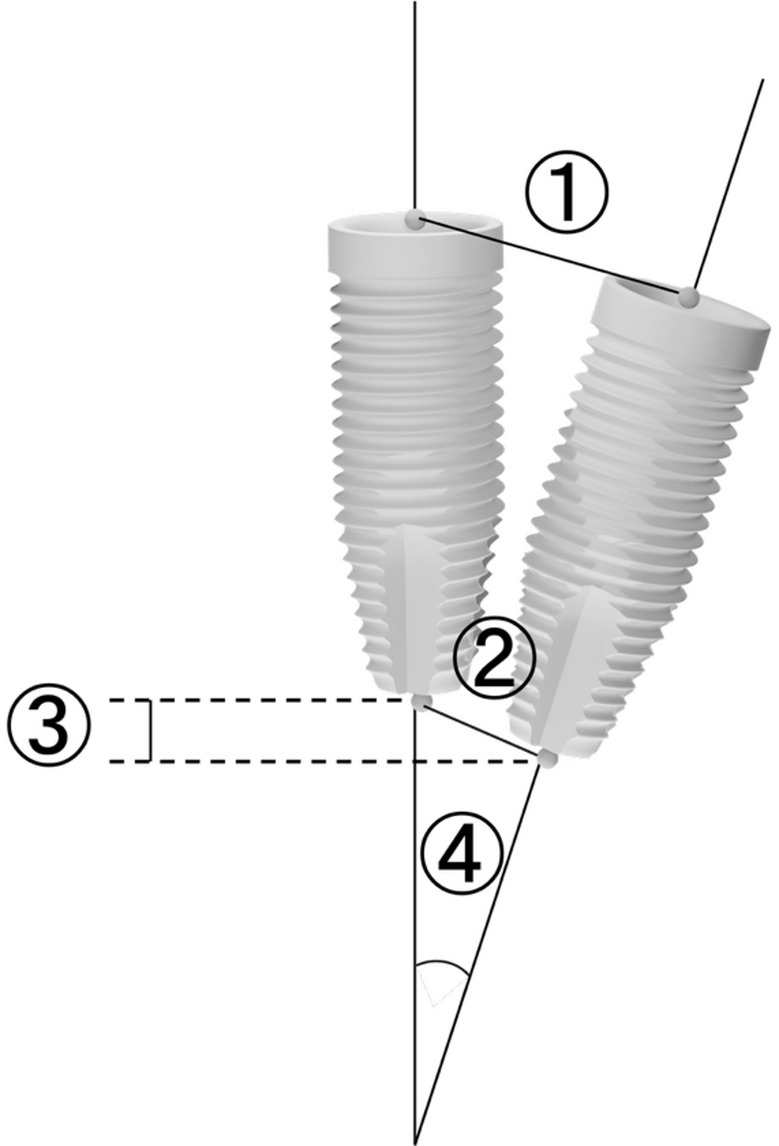
Measurement parameters used to evaluate deviations between the planned and placed implants in d-CAIS. ① Two-dimensional deviation at the implant entry point; ② Three-dimensional deviation at the implant apex; ③ Vertical depth deviation at the implant apex; ④ Angular deviation between the planned and placed implant axes

After implant placement, postoperative CBCT scans were obtained for all models. Implant placement accuracy was assessed by superimposing the preoperative planning data with the postoperative CBCT data. The evaluation parameters included three-dimensional entry point deviation, three-dimensional apex deviation, vertical depth deviation, and angular deviation (Fig. 3). All measurements were performed by a single examiner who was blinded to the group allocation.

### Statistical analysis

In this study, each implant placement was considered an independent observational unit, and all statistical analyses were performed at the implant level (implant-level analysis). Normality of the measured variables was assessed using the Shapiro–Wilk test; however, as normality was not confirmed, group comparisons were consistently performed using the Kruskal–Wallis test. For variables in which the Kruskal–Wallis test indicated statistically significant differences, post hoc analyses were conducted using Dunn’s test with Bonferroni correction. All statistical analyses were performed using Python (statsmodels and SciPy).

## Results

In this study, implant placement accuracy and procedural time achieved with d-CAIS were compared among three patient registration methods (XC, XM, and ND) in a novice operator group. Analyses were performed at the implant level, with n = 25 for each group. Group differences were assessed using the Kruskal–Wallis test, and Bonferroni correction (p × 4) was applied for the four accuracy-related outcome measures. For variables in which the Kruskal–Wallis test indicated significant differences, post hoc pairwise comparisons were conducted using Dunn’s test with Bonferroni correction (p × 3).

### Entry point deviation (2D)

**Fig. 4:**
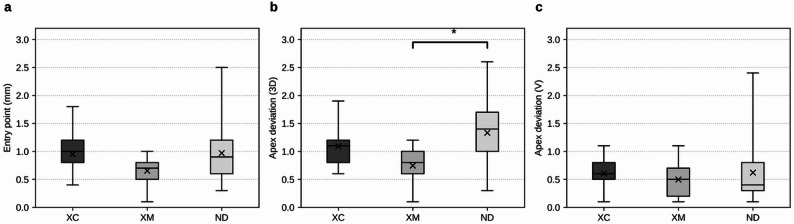
Linear deviations of implant placement accuracy in d-CAIS. Box plots represent the deviations for each group (XC, XM, ND) across the measurement parameters. The lower and upper bounds of each box correspond to the first (Q1) and third (Q3) quartiles, respectively. Horizontal lines within the boxes indicate median values, and “X” markers represent mean values. Whiskers denote the minimum and maximum values within each group. a. Two-dimensional deviation at the implant entry point. b. Three-dimensional deviation at the implant apex. c. Vertical depth deviation at the implant apex

The mean ± standard deviation of two-dimensional entry point deviation was 0.95 ± 0.35 mm (range: 0.4–1.8 mm) in the XC group, 0.68 ± 0.23 mm (range: 0.1–1.0 mm) in the XM group, and 0.97 ± 0.55 mm (range: 0.3–2.5 mm) in the ND group. The median values (Q1/Q3) were 1.0 mm (0.8/1.2) for XC, 0.7 mm (0.6/0.9) for XM, and 0.9 mm (0.6/1.2) for ND. The Kruskal–Wallis test revealed a significant difference among groups (H = 9.20, p = 0.010); however, this difference did not remain statistically significant after Bonferroni correction (adjusted p = 0.040; α = 0.0125). In post hoc analysis using Dunn’s test with Bonferroni correction, a significant difference was observed between the XC and XM groups before correction (p = 0.0045), but this difference was no longer significant after correction (p = 0.0136). No significant differences were detected in any other pairwise comparisons (Fig. 4).

### Apex deviation (3D)

The mean ± standard deviation of three-dimensional apex deviation was 1.09 ± 0.33 mm (range: 0.6–1.9 mm) in the XC group, 0.78 ± 0.33 mm (range: 0.1–1.2 mm) in the XM group, and 1.33 ± 0.57 mm (range: 0.3–2.6 mm) in the ND group. The median values (Q1/Q3) were 1.1 mm (0.8/1.2) for XC, 0.9 mm (0.6/1.0) for XM, and 1.4 mm (1.0/1.7) for ND. The Kruskal–Wallis test demonstrated a statistically significant difference among groups (H = 17.34, p = 0.00017), which remained significant after Bonferroni correction (adjusted p = 0.00069). Post hoc analysis using Dunn’s test with Bonferroni correction revealed a significant difference between the XM and ND groups (adjusted p = 0.00013), with the XM group showing significantly smaller deviations than the ND group. No significant differences were observed between the XC and XM groups (adjusted p = 0.021) or between the XC and ND groups (adjusted p = 0.48) (Fig. 4).

### Apex vertical deviation (V)

The mean ± standard deviation of vertical apex deviation was 0.60 ± 0.28 mm (range: 0.1–1.1 mm) in the XC group, 0.51 ± 0.29 mm (range: 0.1–1.1 mm) in the XM group, and 0.62 ± 0.57 mm (range: 0.1–2.4 mm) in the ND group. The median values (Q1/Q3) were 0.6 mm (0.5/0.8) for XC, 0.5 mm (0.3/0.7) for XM, and 0.4 mm (0.3/0.8) for ND. The Kruskal–Wallis test indicated no significant differences among groups (H = 1.76, p = 0.42), and this result remained non-significant after Bonferroni correction (adjusted p = 1.00). Post hoc analysis using Dunn’s test with Bonferroni correction also revealed no significant differences in any pairwise comparisons (Fig. 4).

### Angular deviation

**Fig. 5:**
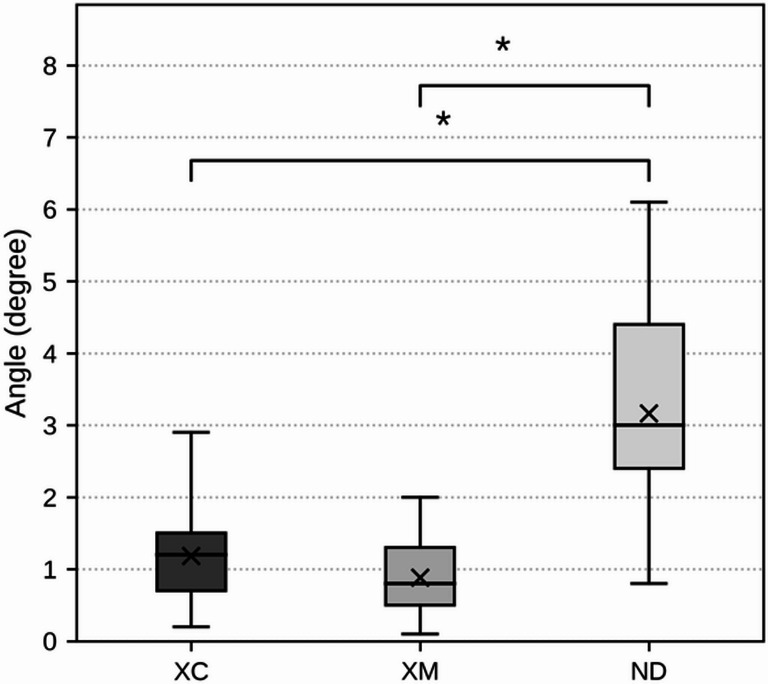
Angular deviation of implant placement among the three registration methods in d-CAIS. Box plots represent angular deviations for each group (XC, XM, ND). The lower and upper bounds of each box correspond to the first (Q1) and third (Q3) quartiles, respectively. Horizontal lines within the boxes indicate median values, and “X” markers represent mean values. Whiskers denote the minimum and maximum values within each group. Statistically significant differences between groups are indicated by significance brackets and asterisks (p < 0.05)

The mean ± standard deviation of angular deviation was 1.18 ± 0.65° (range: 0.2–2.9°) in the XC group, 0.97 ± 0.56° (range: 0.1–2.0°) in the XM group, and 3.16 ± 1.48° (range: 0.8–6.1°) in the ND group. The median values (Q1/Q3) were 1.2° (0.7/1.5) for XC, 0.9° (0.5/1.4) for XM, and 3.0° (2.4/4.4) for ND. The Kruskal–Wallis test demonstrated a statistically significant difference among groups (H = 33.21, p = 6.14 × 10^−^⁸), which remained significant after Bonferroni correction (adjusted p = 2.46 × 10^−^⁷). Post hoc analysis using Dunn’s test with Bonferroni correction showed that the ND group exhibited significantly greater angular deviation than both the XC group (adjusted p = 0.000028) and the XM group (adjusted p = 0.00000019). No significant difference was observed between the XC and XM groups (adjusted p = 0.99) (Fig. 5).

### Procedural time

**Fig. 6:**
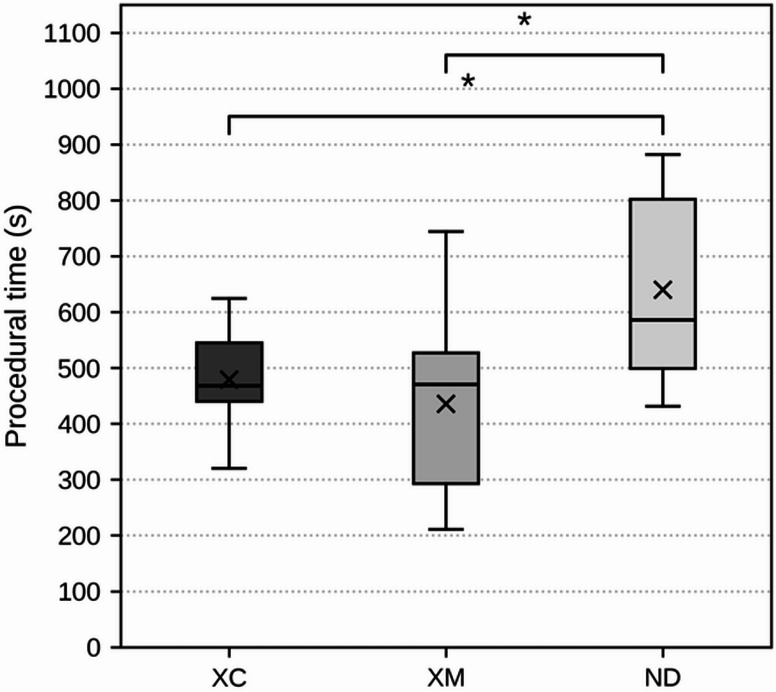
Procedural time required for implant placement among the three registration methods in d-CAIS. Procedural time was defined as the duration from completion of patient registration to final implant placement. Box plots represent procedural time for each group (XC, XM, ND). The lower and upper bounds of each box correspond to the first (Q1) and third (Q3) quartiles, respectively. Horizontal lines within the boxes indicate median values, and “X” markers represent mean values. Whiskers denote the minimum and maximum values within each group. Statistically significant differences between groups are indicated by significance brackets and asterisks (p < 0.05)

The mean ± standard deviation of procedural time was 478.88 ± 66.5 s (range: 320–624 s) in the XC group, 435.24 ± 146.7 s (range: 211–744 s) in the XM group, and 640.00 ± 163.0 s (range: 431–882 s) in the ND group. The median values (Q1/Q3) were 474 s (441/597 s) for XC, 447 s (291/530 s) for XM, and 586 s (503/770 s) for ND. The Kruskal–Wallis test revealed a statistically significant difference among the three groups (H = 46.78, p = 6.92 × 10^−11^). Post hoc analysis using Dunn’s test with Bonferroni correction demonstrated that the ND group required significantly longer procedural times than both the XC group (adjusted p = 3.96 × 10^−^⁸) and the XM group (adjusted p = 1.53 × 10^−11^). No significant difference was observed between the XC and XM groups (adjusted p = 0.75) (Fig. 6).

## Discussion

The present study investigated the effects of three patient registration methods on implant placement accuracy and procedural time in d-CAIS when performed by novice operators. The results demonstrated significant differences among registration methods in three-dimensional apex deviation and angular deviation, with the ND group exhibiting significantly greater angular deviation and longer procedural time [21, 26, 27]. In contrast, no significant differences were observed among groups for entry point deviation or vertical apex deviation [25, 28].

In the present study, no statistically significant intergroup differences were observed for entry point deviation or vertical apex deviation after Bonferroni correction, regardless of the registration method. This finding indicates that, even among novice operators, the real-time feedback provided by d-CAIS enables relatively stable control of the initial implant position and insertion depth [29, 30]. Notably, vertical apex deviation represents a critical parameter directly related to clinical safety and prosthetic planning. The observation that stable depth control was achieved independently of the registration method in novice operators supports the educational value of d-CAIS [31]. Taken together, these results clearly demonstrate that, in novice operators, d-CAIS can strongly support fundamental aspects of implant placement—specifically, determining where to start and how deep to place the implant—irrespective of the registration strategy used [19–21, 26, 27].

With respect to angular deviation, this study demonstrated the most pronounced intergroup differences, with the ND group exhibiting significantly greater angular deviation than both the XC and XM groups. Angular control during d-CAIS strongly depends on the integration of visual feedback and three-dimensional spatial perception, and this parameter has been reported to be particularly unstable among novice operators [21, 27]. The increased angular deviation observed in the ND group may be attributable not only to variability associated with dental surface tracing during surface-based registration, but also to characteristics of the tracker fixation design used in ND systems. In ND, a wire-based arm is employed to support the patient-side tracker. While this structure provides a high degree of adjustability, it may also be more susceptible to subtle deflection or vibration induced by intraoperative manipulation or external forces [32–34]. In novice operators, compensatory maneuvers or judgments required to address such minor tracker instability may be challenging, potentially allowing tracking errors to manifest as increased angular deviation [35, 36].

Regarding procedural time, the ND group required significantly longer times than both the XC and XM groups, whereas no significant difference was observed between the XC and XM groups. In this study, procedural time was defined as the duration from completion of patient registration to final implant placement and did not include the time required for the registration process itself. In this context, the prolonged procedural time observed in the ND group may be attributable not to the registration workflow per se, but rather to the reliability of tracking and navigation display during implant placement. Specifically, subtle movement associated with the wire-based arm used for tracker fixation may have affected tracking stability, potentially rendering the navigation display less reliable for novice operators [37]. As a consequence, operators may have needed to repeatedly verify angulation and position during placement, leading to an extension of the pure implant insertion time [35, 38, 39]. Importantly, the longer procedural time observed in the ND group was not accompanied by deterioration in entry point deviation or vertical apex deviation. This finding suggests that, even when novice operators perceived instability in navigation feedback, they may have compensated through more cautious manipulation to correct position and depth, indicating that procedural time and placement accuracy are not necessarily directly correlated.

The findings of the present study indicate that, even among novice operators, the use of d-CAIS enables stable accuracy in entry point positioning and insertion depth regardless of the registration method employed. In contrast, angular control and procedural efficiency appear to be more susceptible to the influence of registration workflows, tracker design, and characteristics of the navigation display [40, 41]. These observations suggest that, for the educational implementation of d-CAIS, it is insufficient to merely introduce navigation systems into training programs. Rather, effective training should incorporate a thorough understanding of tracking mechanisms and navigation display characteristics, as well as their potential impact on intraoperative decision-making and motor control [19, 20, 23].

This study has several limitations. First, it was conducted as an in vitro model-based experiment, and therefore did not account for clinical factors such as soft tissue conditions, bleeding, or patient movement encountered in actual surgical settings. Consequently, caution is required when extrapolating the present findings directly to clinical practice. Second, statistical analyses were performed by considering each implant placement as an independent observational unit, and intra-operator correlation was not explicitly incorporated into the statistical models. However, because the primary objective of this study was to evaluate variability during the learning phase of novice operators, the use of implant-level analysis was considered reasonable. Third, the procedural time evaluated in this study was defined as the duration from completion of registration to final implant placement and did not include the time required for the registration process itself. Future studies should incorporate more comprehensive time assessments, including registration duration and overall procedural workload.

## Conclusion

This study investigated the effects of three patient registration methods on implant placement accuracy and procedural time in d-CAIS performed by novice operators. The results demonstrated that, even among novice operators, stable accuracy in entry point positioning and insertion depth can be achieved regardless of the registration method used. In contrast, angular deviation and procedural time were influenced by differences in registration workflows and system characteristics, with the ND group showing significantly greater angular deviation and longer procedural times.

These findings suggest that, while d-CAIS can strongly support fundamental aspects of implant placement—such as position and depth control—in novice operators, angular control and procedural efficiency are more susceptible to the characteristics of the registration method, tracking mechanism, and navigation display. The insights gained from this study may provide valuable guidance for the educational implementation of d-CAIS, the design of training environments for beginners, and system selection during the early stages of clinical adoption.

## Data Availability

The datasets generated and/or analyzed during the current study are available from the corresponding author upon reasonable request.
